# Adaptive geostatistical sampling enables efficient identification of malaria hotspots in repeated cross-sectional surveys in rural Malawi

**DOI:** 10.1371/journal.pone.0172266

**Published:** 2017-02-14

**Authors:** Alinune N. Kabaghe, Michael G. Chipeta, Robert S. McCann, Kamija S. Phiri, Michèle van Vugt, Willem Takken, Peter Diggle, Anja D. Terlouw

**Affiliations:** 1 Center of Tropical Medicine and Travel Medicine, Department of Infectious Diseases, Division of Internal Medicine, Academic Medical Center, University of Amsterdam, Amsterdam, Netherlands; 2 Public Health Department, College of Medicine, University of Malawi, Blantyre, Malawi; 3 Lancaster Medical School, Lancaster University, Lancaster, United Kingdom; 4 Malaria Theme, Malawi-Liverpool Wellcome Trust, Blantyre, Malawi; 5 Laboratory of Entomology, Wageningen University and Research, Wageningen, Netherlands; Tulane University School of Public Health and Tropical Medicine, UNITED STATES

## Abstract

**Introduction:**

In the context of malaria elimination, interventions will need to target high burden areas to further reduce transmission. Current tools to monitor and report disease burden lack the capacity to continuously detect fine-scale spatial and temporal variations of disease distribution exhibited by malaria. These tools use random sampling techniques that are inefficient for capturing underlying heterogeneity while health facility data in resource-limited settings are inaccurate. Continuous community surveys of malaria burden provide real-time results of local spatio-temporal variation. Adaptive geostatistical design (AGD) improves prediction of outcome of interest compared to current random sampling techniques. We present findings of continuous malaria prevalence surveys using an adaptive sampling design.

**Methods:**

We conducted repeated cross sectional surveys guided by an adaptive sampling design to monitor the prevalence of malaria parasitaemia and anaemia in children below five years old in the communities living around Majete Wildlife Reserve in Chikwawa district, Southern Malawi. AGD sampling uses previously collected data to sample new locations of high prediction variance or, where prediction exceeds a set threshold. We fitted a geostatistical model to predict malaria prevalence in the area.

**Findings:**

We conducted five rounds of sampling, and tested 876 children aged 6–59 months from 1377 households over a 12-month period. Malaria prevalence prediction maps showed spatial heterogeneity and presence of hotspots—where predicted malaria prevalence was above 30%; predictors of malaria included age, socio-economic status and ownership of insecticide-treated mosquito nets.

**Conclusions:**

Continuous malaria prevalence surveys using adaptive sampling increased malaria prevalence prediction accuracy. Results from the surveys were readily available after data collection. The tool can assist local managers to target malaria control interventions in areas with the greatest health impact and is ready for assessment in other diseases.

## Introduction

In the context of malaria elimination, limited resources, significant decline in malaria incidence and prevalence [1, 2], interventions will need to target high disease burden areas to further reduce transmission [3–5]. Malaria exhibits spatial and temporal heterogeneity in both stable and endemic transmission settings [4, 6]. Current tools for monitoring or reporting malaria burden lack the capacity to detect high malaria transmission areas, often called “hotspots”, and to report continuous changes of disease burden over time [7]. National malaria control programmes rely on national surveys such as Malaria Indicator Surveys (MIS) and Demographic and Health Surveys (DHS), or use health facility malaria case reports and/or registers to monitor malaria burden and the progress of malaria control [8]. The surveys generally are cross sectional, use random population samples, do not report real-time results and are repeated after long periods of time (at least two years). These routine surveys lack fine scale spatial heterogeneity information for malaria prevalence, and only produce data at national and regional level rather than sub district level. In limited-resource settings, facility case registers, where available, provide unreliable data [9, 10], under-represent the burden of disease in the community, are incomplete, prone to errors, and may misreport the number of cases due to lack of diagnostic capacity [9, 11–13]. Although District Health Information System (DHIS2) allows mapping of disease burden and intervention coverage at regional and district levels based on the routine data, precise geolocation are unavailable at sub-district level and also, the available data depend on proportion of the community utilising the health services.

Continuous disease surveys allow continuous monitoring of changes in spatial and temporal disease distribution at national, regional and district levels; the surveys are potential tools to accurately monitor disease control progress in low resource settings where surveillance systems are weak [14]. Continuous malaria prevalence surveys allow continuous analysis of data, mapping of malaria prevalence, and reporting short term changes in disease prevalence and intervention coverage [15]. Monthly cross sectional prevalence surveys report results within a short duration [16]. Use of such surveys would assist district managers to identify high disease burden areas (“hotspots”) for early targeted intervention [3].

Recent developments in geostatistical modelling offer opportunities to develop more accurate predictive methods for disease burden [17, 18]. Geostatistical modelling can be used to map disease risk and visualise spatial and temporal changes of disease burden and intervention coverage. The random sampling of clusters used currently in surveys, lacks the accuracy for detecting fine scale spatial heterogeneity of disease burden. These sampling methods may under-represent heterogeneously distributed and hard to reach populations in limited resource settings [19]. An adaptive geostatistical design (AGD), would allow gain in statistical sampling efficiency by focusing on areas where prediction of the measure of interest is imprecise. AGD allow sampling to focus on sub-regions where precise prediction is needed to inform public health action. Chipeta et al. [20] previously demonstrated AGD on simulated data and reported potential for improved prediction of malaria prevalence compared to non-adaptive (random) sampling.

We describe the first field application of AGD sampling in continuous malaria prevalence surveys for a 12 month period, and we present malaria prevalence maps from the study site in Chikwawa district, Malawi.

## Materials and methods

### Study setting

**Fig 1:**
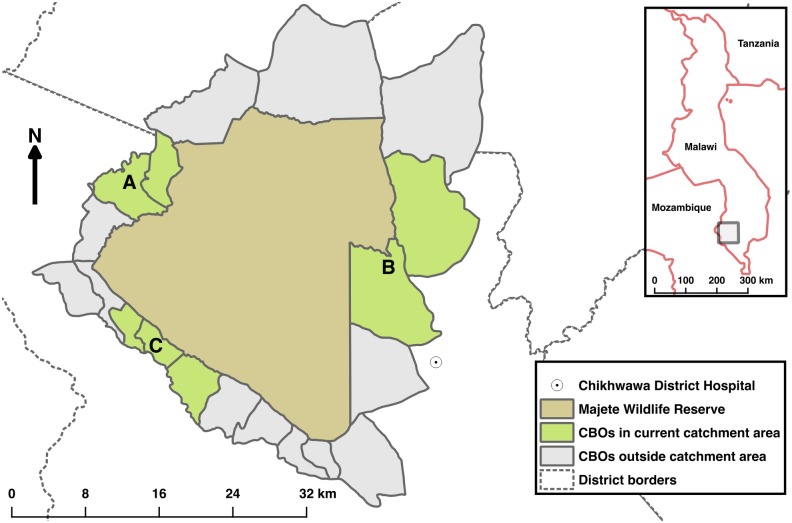
Map of Majete wildlife reserve and surrounding communities. Majete Wildlife Reserve (brown) is surrounded by 19 community based organisations—CBOs (grey and green) comprising the Majete perimeter. Three focal areas (green), labelled as A, B, and C mark the communities selected for malaria indicator surveys. The rest of the CBOs (grey) are outside the projects catchment area.

We conducted the study in villages surrounding Majete Wildlife Reserve (MWR) in Chikwawa district, southern Malawi from April 2015 to April 2016. Malaria transmission is intense and peaks from December to March during the rainy season [21]. The study area is within the catchment of the Majete Malaria Project (MMP), a five-year, community-based malaria control project. The surveys were conducted in 61 villages with approximately 6,600 households, and a total population of approximately 25,000. The area was divided into three administrative units, which, for convenience purposes are referred to as *focal areas*: A, B and C; see Fig 1 from which villages and households within villages were sampled.

### Study design

The sampling unit in the study was the household. We used repeated cross-sectional household surveys for malaria parasitaemia (rolling Malaria Indicator Survey—rMIS) data guided by AGD. rMIS involves collecting and analysing data from a sampled set of households, then repeating the process for subsequent sampled sets of households. In this design, on any sampling occasion, the choice of sampling households was informed by prevalence results from spatial predictive modelling of data collected on earlier occasions; a different set of households was chosen on each occasion from locations where uncertainty of the estimate was highest. The adaptive design problem consisted of deciding which households to sample in each round of sampling to optimise the precision of the resulting sequence of area-wide prevalence maps. Note that each household that meets the AGD constraints has a non-zero probability of being sampled at each round of sampling. Also, once a household was sampled in a round, it was excluded from subsequent sampling frame and hence subsequent sampling rounds.

### Participants

We invited children 6–59 months and women 15–49 years old who slept in the sampled household the previous night to participate. If the head of household consented we interviewed, tested for malaria and anaemia and recorded temperature, weight, height and mid upper arm circumference measurements. Households that did not have any eligible participants were only interviewed; no clinical assessment or blood tests were done.

### Procedures

We developed electronic forms and training material adapted from the global malaria indicator survey toolkit [22]. Research teams comprising a research nurse and 2 to 4 research assistants invited sampled household members to central locations where consent was obtained from the head of household. Teams followed up household members who did not present to the central location to conduct the survey. Sampled households that were unoccupied, or had been demolished, were replaced by the nearest household. The teams administered a questionnaire and tested eligible participants for malaria and anaemia using a rapid diagnostic test (RDT; SD Bioline Ag P.f (HRP)) and hemocue 301 (Haemocue, Angelholm, Sweden), respectively. Participants with RDT positive results or low hemoglobin reading (less than 11g/dl) were managed according to Malawi national treatment guidelines or referred to a health facility, respectively.

### Household sampling

**Fig 2:**
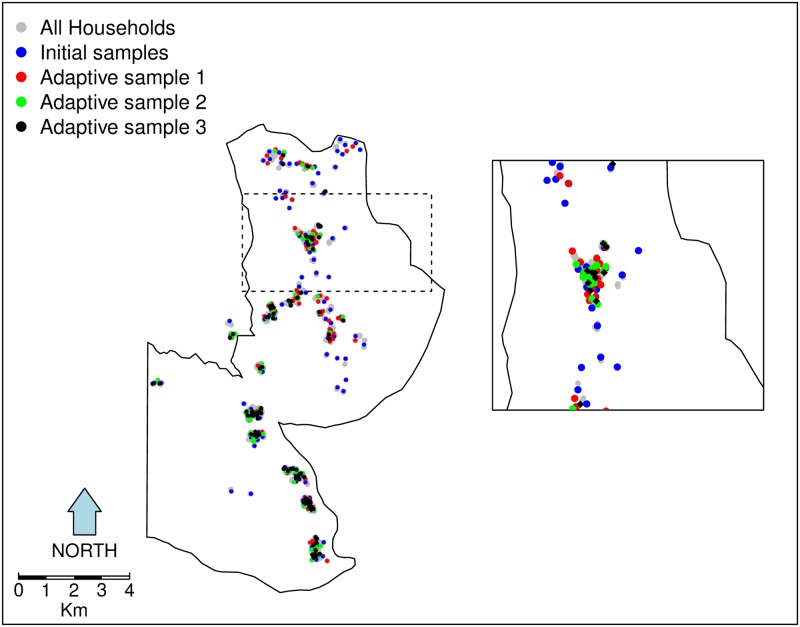
Adaptive sampling in practice, initial spatially inhibitory design samples augmented with adaptive design samples. The map illustrates adaptive sampling in practice. All households in the study area are shown as grey dots. Results from the initial inhibitory random samples households (blue dots) and subsequent samples were used to generate the next adaptive samples (red, green and black dots). Each subsequent sample, used accruing data from previous sample results. Inset shows a zoomed-in subset of locations.

The first stage in the geostatistical design of the study was a complete enumeration of households in the study region from August to November 2014; Geo-location data were collected using Global Positioning System (GPS) devices on Samsung Galaxy Tab 3 running Android 4.1 Jellybean Operating System, accurate to within 5 meters on open data kit (ODK) platform. We used the enumeration data to sample the first 100 households in each of the three focal areas using a spatially inhibitory random sampling design [23], to achieve approximately uniform coverage of each of the focal areas in the study-area. The second round of sampling also followed a spatially inhibitory sample. At the end of these two initial and each subsequent sampling period, a standard operating procedure was followed in checking data for consistency and completeness before uploading them to an off-site database server. The accumulating data up to that period were analysed immediately and the prevalence prediction results fed into an adaptive sampling algorithm to inform the choice of new sampling locations in the next sampling round (after excluding already sampled households). We sampled 90 households per two months per focal area in each of the subsequent sampling rounds. Fig 2 shows a map of focal area B with an inset to demonstrate adaptive sampling in practice. Adaptive geostatistical designs are explained in more details by Chipeta et al [20].

### Statistical analysis

The primary outcome from each individual was a binary indicator for a positive or negative malaria test by malaria RDT in children aged 6–59 months. Malaria transmission hotspots were areas with predicted parasitaemia prevalence above 30% in children aged 6–59 months; the national malaria RDT prevalence estimate in this age-group was 37.1% in Malawi MIS 2014 [8]. Age of each individual, availability of at least one ITN and socio-economic status (SES) were considered, as defined in the results. For SES, an indicator of household wealth taking discrete values from 1 (poor) to 5 (wealthy), was derived by an application of principal component analysis as discussed in Vyas et al [24]. Data for elevation were derived using the Advanced Space-borne Thermal Emission and Reflection Radiometer Global Digital Elevation Model (ASTER GDEM) version 2, which has a spatial resolution of 30 meters. The data were downloaded from the United States Geological Survey (USGS, http://gdex.cr.usgs.gov/gdex/). Normalised difference vegetation index (NDVI) data were calculated based on images from the Landsat 8 satellite, also downloaded from the USGS (http://earthexplorer.usgs.gov/). For NDVI measure, we calculated and used mean values for the above sampling period. Data from all rounds were combined for this analysis, such that we did not account for seasonal differences across the year; this was due to few sampling rounds within the study duration.

We implemented estimation and predictive modelling in a Bayesian framework, using Bayesian geostatistical binary probit model. The model allows specifying a two-level model to include individual-level and household-level (or any other unit comprising a group of individuals) variables. We used the geostatistical binary probit model for binary response data in the following manner. Let *i* and *j* denote the indices of the *i*-th household and *j*-th individual within that household. The response variable *Y*_*ij*_ is a binary indicator taking value 1 if the individual has been tested positive for malaria and 0 otherwise. Conditionally on a zero-mean stationary Gaussian process *S(x*_*i*_*)*, *Y*_*ij*_ are mutually independent Bernoulli variables with probit link function Φ^−1^(·),

Yij|dij, S(xi) ~ Bernoulli(pij)

Φ−1(pij) = d′ijβ + S(xi) (1)

where *d*_*ij*_ is a vector of covariates, both at individual- and household-level, with associated regression coefficients. For details see Rue and Held [25]; Beron and Vijverberg [26]; Berrett and Calder [27]. The Gaussian process *S(x)* has isotropic Matern covariance function [28] with variance *σ*^*2*^, scale parameter *φ* and shape parameter *κ*.

The target for predictive inference is *T* = *T*(*S*), i.e. malaria prevalence prediction for unobserved locations in the study region. Additionally, we delineate sub-regions of the study region where prevalence *p(x)* is likely to exceed a policy intervention/national threshold, exceedence probability, in which case the target becomes *T* = {*x*: *p*(*x*) > ***c***} for pre-specified ***c***. All analyses were done in R statistical environment version 3.3.0 [29].

### Ethical consideration

Ethical clearance for the study was obtained from the University of Malawi, College of Medicine Research Ethics Committee (COMREC) in Malawi (P.09/14/1631). Permissions were obtained from the Ministry of Health and the district health authorities in Chikwawa District. Prior to the start of the study, a series of meetings were held in participating communities to explain the nature and purpose of the study. We obtained individual written informed consent and in the case of children, from their parents or legal guardians.

## Results

We conducted five sampling rounds within 12 months and completed data-collection from 1,377 (87.8%) of the 1,568 sampled households (Table 1). Consent was refused from 41 (2.6%) households. Data-collection was not completed in a further 149 (9.5%) households, mainly because the house was vacated between the initial enumeration and the time of household sampling. From the total sampled households, 1,044 (67.5%) had either children 6–59 months, women 15–49 years or both eligible children and women. A total of 876 children aged 6–59 months were tested for malaria and anaemia; we excluded results from women of child-bearing age in the analysis as malaria prevalence surveys are based on children. It took an average of 4–8 weeks to complete data collection per sampling round; results of each sampling round were available within 1–2 weeks after completion of data collection and cleaning.

**Table 1:** Characteristics for sampled households within Majete wildlife reserve perimeter.

	*N*	*%*
**Total sampled households**	1,568	-
**Households completed**	1,377	87.8
**Refused consent**	41	2.6
**Children 6–59 months in sampled households**	1,016	
**Children 6–59 months enrolled**	876	86.2*
**Household wealth quintile**		
** Lowest**	390	28.3
** Second**	196	14.2
** Middle**	258	18.7
** Fourth**	267	19.4
** Top**	266	19.3

* Percentage of eligible children from sampled households who actually took part in the survey.

For covariate selection we used ordinary probit regression, retaining covariates with nominal *p*-values less than 0.05 (S1 Table); these ignore the effects of spatial correlation and are likely to be anti-conservative, thereby avoiding false exclusion of potentially important covariates. This resulted in the set of covariates shown in Table 2, with terms for social economic status (SES), availability of at least one ITN, NDVI, and elevation. The *σ*^*2*^ and *φ* are variance of the Gaussian process and scale of the spatial correlation respectively. We then fitted the geostatistical binary probit model (1) to obtain the Bayesian estimates of the parameters and associated 95% highest posterior density (HPD), as also shown in Table 2. Each evaluation of the Markov chain Monte Carlo used 2,000 simulated values, obtained by conditional simulation of 21,000 values and sampling every 10th realisation after discarding a burn-in of 1,000 values.

**Table 2:** Bayesian estimates and 95% highest posterior density intervals for the model fitted to the Majete malaria data for children 6–59 months.

Term	Estimate	95% HPD
**Intercept**	0.6647	(0.1538, 1.0850)
**SES**	-0.0737	(-0.1087, -0.0337)
**ITN**	-0.1829	(-0.3166, -0.0337)
**Age**	-0.4921	(-0.6045, -0.3903)
**Elevation**	-0.0009	(-0.0015, -0.0004)
**NDVI**	0.0524	(-1.1358, 0.9811)
***σ*^*2*^**	0.4693	(0.2154, 0.8109)
***φ***	2.3869	(0.7629, 4.9778)

HPD = Highest Posterior Density, ITN = Insecticide-Treated Net (availability of at least one in household), NDVI = Normalised Difference Vegetation Index, SES = Social Economic Status.

From Table 2, an increase in SES, age and ownership of at least one ITN are all associated with a reduction in the probability of a positive RDT. Elevation was negatively associated with probability of a positive RDT whereas NDVI shows a positive, but non-significant, association.

**Fig 3:**
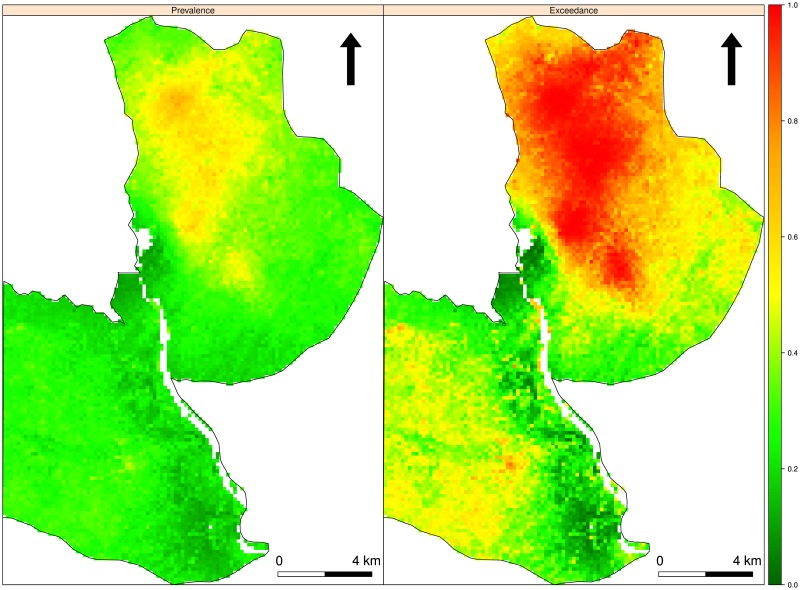
Malaria prevalence and exceedance probabilities maps. Left panel shows malaria prevalence in children 6–59 months in focal area B. The right-hand panel shows the map of exceedance probabilities *P(x; 0*.*3)* for the Bayesian prediction.

**Fig 4:**
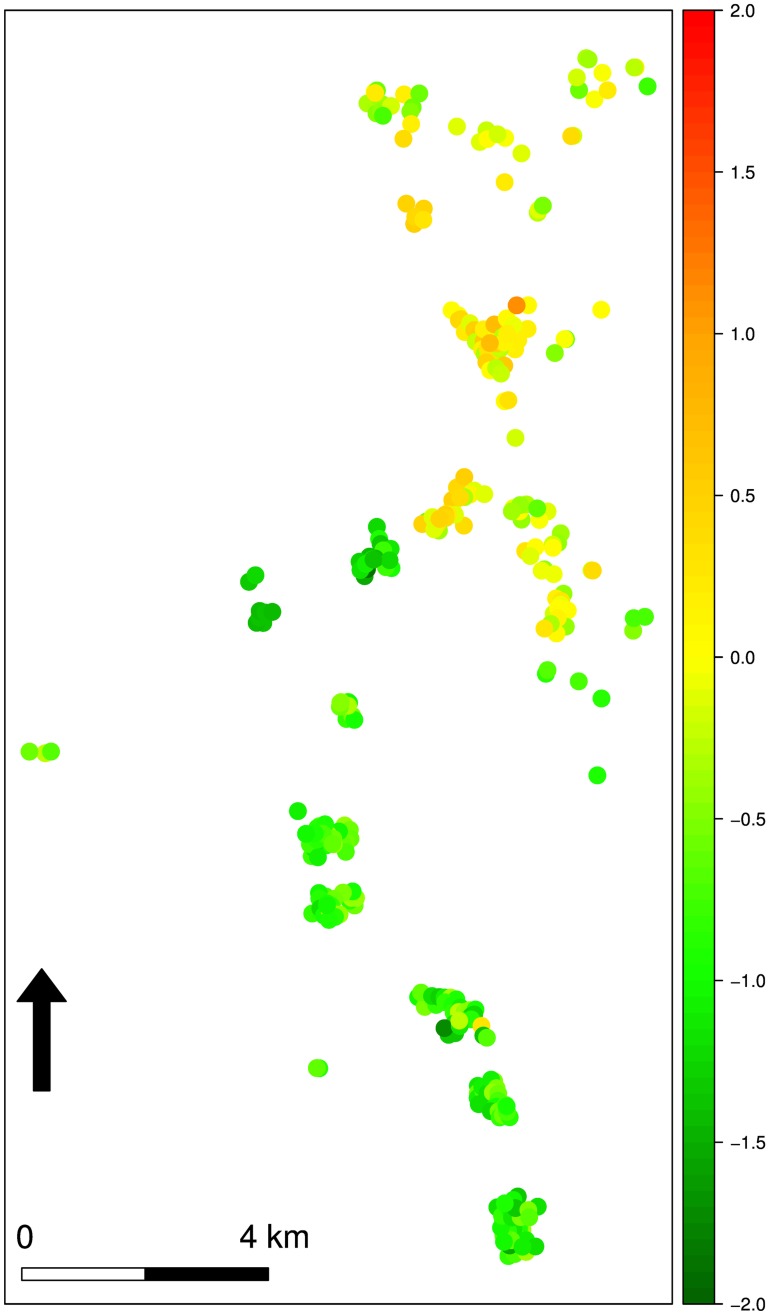
Unexplained spatial variation map. Contributions of the linear regression and of the unexplained spatial variation to the predicted log-odds of malaria prevalence in children 6–59 months at each of the observed locations in focal area B.

Here, we present maps of malaria prevalence in children aged 6–59 months in focal area B. Prevalence maps for focal areas A and C are provided in the supplementary material (S2 and S3 Figs). Overall, prevalence is higher in focal area B compared to focal areas A and C; however, Fig 3 (left panel) shows that prevalence is generally low in the south-west of the region, whereas the north-east has pockets of comparatively high malaria prevalence. Hotspots in focal areas A and C are mainly localised (S2 and S3 Figs). Fig 3 (right panel) shows the map of exceedance probabilities that prevalence is over the national threshold of 30%. Fig 4 shows the contributions of the linear regression to the predicted log-odds of prevalence at each of the observed locations in focal area B.

## Discussion

We have modelled malaria prevalence in children aged 6–59 months in a rural area of southern Malawi using individual, household, and environmental data as covariates, and allowing for spatial correlation. Adaptive sampling prior to each round of data collection was used to identify areas where increased sampling effort should be focused to maximise the increase in overall predictive accuracy. Malaria prevalence predictions at observed locations show disease burden at the finest scale possible, and we detected multiple malaria hotspots across the study regions. To our knowledge this is the first time an adaptive sampling technique has been implemented to monitor spatial distribution of malaria or any disease in a human population.

Other studies map disease prevalence heterogeneity using national and community surveys (conducted at different time points), expert opinion, facility data or a combination of these data sources [30–34]. With an adaptive sampling technique, we avoided reporting results based on multiple data sources which differ in accuracies, collection times, and sampled areas. Health facility disease registers in resource limited settings contain low quality, incomplete and unreliable data [9, 11, 13]. These data are inadequate to monitor fine changes in spatial and temporal malaria prevalence variations. Using continuous surveys based on AGD readily provides results of representative cross-sectional surveys soon after data collection. The continuous surveys monitor short-term spatial and temporal changes of disease burden to enable managers to detect and target areas that need scaling up of interventions. The uptake and impact of malaria control interventions can also be monitored.

Compared to the recommended national MIS, the continuous prevalence surveys using AGD are not as logistically demanding. The surveys can potentially be conducted by district personnel throughout a prolonged period to complement the 2-yearly MIS. The actual data collection required small teams and took a short period of time to complete. Cost effectiveness of implementing continuous surveys using AGD will be assessed and discussed in a separate paper, though a previous study in the same geographic area reported continuous malaria surveys using random sampling was affordable and logistically simple compared to national MIS [16].

The current recommended 2-yearly national MIS are cross sectional surveys using a two stage sample design based on geographical clusters known as enumeration areas. The sampling process is: a) random probability sampling of clusters, b) household enumeration of sampled clusters, c) then random probability sampling of households in the sampled clusters. Cluster sampling under-represents disease burden for heterogeneously distributed diseases and hard to reach populations [19]. The national MIS reports univariate malaria prevalence at national or regional level and without a confidence interval; the surveys are not designed to produce estimates at district or sub-district levels. Comparing disease prevalence between surveys would be inaccurate as sampled points are different and the proportions are crude (unadjusted without confidence intervals). Furthermore, the national MIS reports data from a single time point though malaria prevalence exhibits spatial and temporal variations.

By combining AGD and continuous malaria prevalence surveys, we maximise the precision of malaria prevalence predictions at local level. Adaptive samples add value to continuous prevalence surveys. Rather than continuously selecting random samples, subsequent samples depend on previous prevalence results calculated from contributions of individual, household and environmental predictors; this allows for models to be refined as data becomes available. The subsequent samples focus on areas of relatively high uncertainty to enable more precise delineation of areas where disease prevalence is above or below a given threshold ***c*;** for example, predictive probabilities of the exceedance of policy relevant or national thresholds. AGDs also provide a more complete picture of spatial variations [20]. This approach can potentially empower both local and national programme managers to invest limited resources and efforts on high priority areas for elimination [3–5, 16].

Our innovative approach for the discovery of malaria hotspots can be further fine-tuned by estimates of Plasmodium transmission intensities through monitoring of mosquito populations. The combined result is instrumental for effective application of malaria interventions [3].

We demonstrate the first application of adaptive sampling for continuous spatial diseases surveillance in this small study population. This approach is ideal for measuring disease heterogeneity at sub-district and potentially district levels. A significant challenge in implementing rMIS using AGD at a larger scale (national or regional levels) in most resource-limited settings is the unavailability of geo-referenced households for the sampling frame. In our study, all households were enumerated and geo-referenced to create the sampling frame. At national level, national MIS use a 2-stage cluster sampling approach based on clusters from most recent population census. Similarly, if practical constraints dictate a 2-stage sampling, then AGD sampling must be done in the second stage, within each sampled cluster. Using a cluster as a sampling unit, a national or regional AGD rMIS potentially measures disease heterogeneity, and identifies hotspots at cluster level. This then guides household AGD rMIS. Large scale implementation also requires technical expertise to manage data collection, analysis, and the continuous sampling process.

Algorithms are being developed and will be available as an R package on the comprehensive R archive network (CRAN) website. The modules can be developed for real time monitoring of disease prevalence. For example, the Meningitis Environmental Risk Information Technologies (MERIT) initiative developed such a module for meningitis epidemics prediction [35, 36].

AGD enables more efficient estimation of spatial variation than traditional simple random sampling strategies [20], whilst retaining the objectivity of probability-based sampling. In AGD the initial sample is a probability sample [20], albeit one that is restricted to induce a degree of spatial regularity into sampled locations, and therefore achieves its increase in efficiency without risk of introducing subjective bias.

The repeated cross-sectional AGD methods are generally versatile and may apply to diseases with similar heterogeneity patterns [37, 38]. For example, high disease burden for neglected tropical diseases areas such as onchocerciasis, schistosomiasis etc. can be identified and targeted for interventions such as mass drug administration.

## Conclusion

AGD are automated algorithms that help in sampling optimisation decisions for prevalence surveys. Applying AGD to continuous disease surveys provides fine-scale disease prevalence prediction in limited-resource settings and can be a reliable surveillance tool for both district and national level programme managers. AGD results were readily available during the survey and identified several hotspots in each of the focal areas. This disease monitoring approach is ready to be assessed at a larger scale and for other diseases.

## Supporting information

S1 Fig Map of malaria prevalence and exceedance probability for focal area A. The top panel show malaria prevalence in children 6–59 months in focal area A. The bottom panel shows the map of exceedance probabilities *P(x; 0*.*3)* for the Bayesian prediction.
(TIF)

S2 Fig Map of malaria prevalence and exceedance probability for focal areas C. The top panel show malaria prevalence in children 6–59 months in focal area C. The bottom panel shows the map of exceedance probabilities *P(x; 0*.*3)* for the Bayesian prediction.
(TIF)

S3 Fig Unexplained spatial variation map for focal area A. Contributions of the linear regression and of the unexplained spatial variation to the predicted log-odds of malaria prevalence in children 6–59 months at each of the observed locations in focal area A.
(TIF)

S4 Fig Unexplained spatial variation map for focal area C. Contributions of the linear regression and of the unexplained spatial variation to the predicted log-odds of malaria prevalence in children 6–59 months at each of the observed locations in focal area A.
(TIF)

S1 File Data. CSV file containing data used in the analysis.
(CSV)

S1 Table Parameter estimates from non-spatial probit model for malaria prevalence in children 6–59 months. ITN: insecticide treated bed nets, NDVI: normalized difference vegetation index, SES: socio-economic status.
(DOCX)
