# Supplementary figures and images for: Adaptive geostatistical sampling enables efficient identification of malaria hotspots in repeated cross-sectional surveys in rural Malawi
Source: PLoS One. 2017 Feb 14;12(2):e0172266. doi: 10.1371/journal.pone.0172266 (PMC5308819; doi:10.1371/journal.pone.0172266)

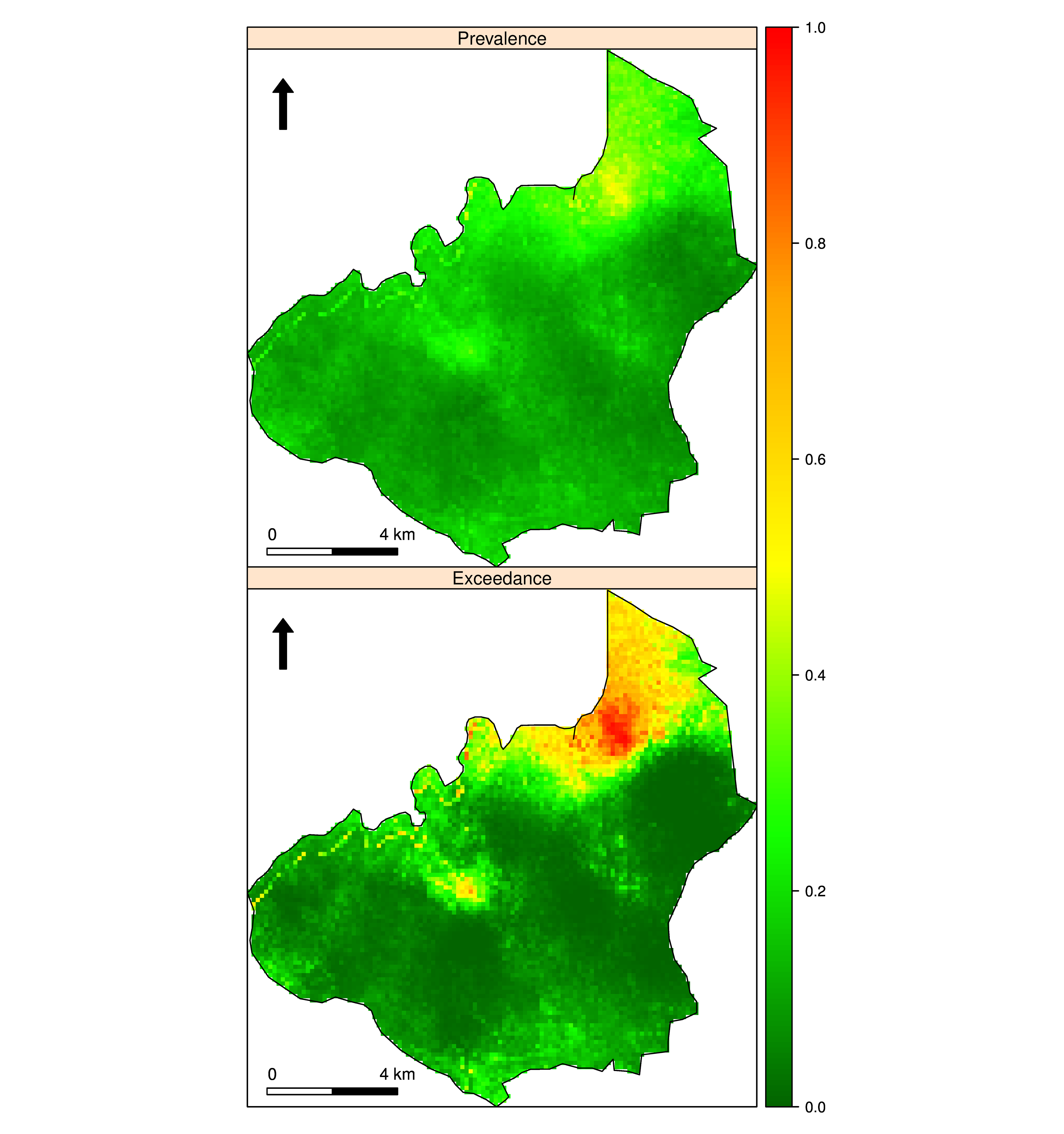

Supplement: S1 Fig — The top panel show malaria prevalence in children 6–59 months in focal area A. The bottom panel shows the map of exceedance probabilities P(x; 0.3) for the Bayesian prediction. (TIF) [file pone.0172266.s001.tif]

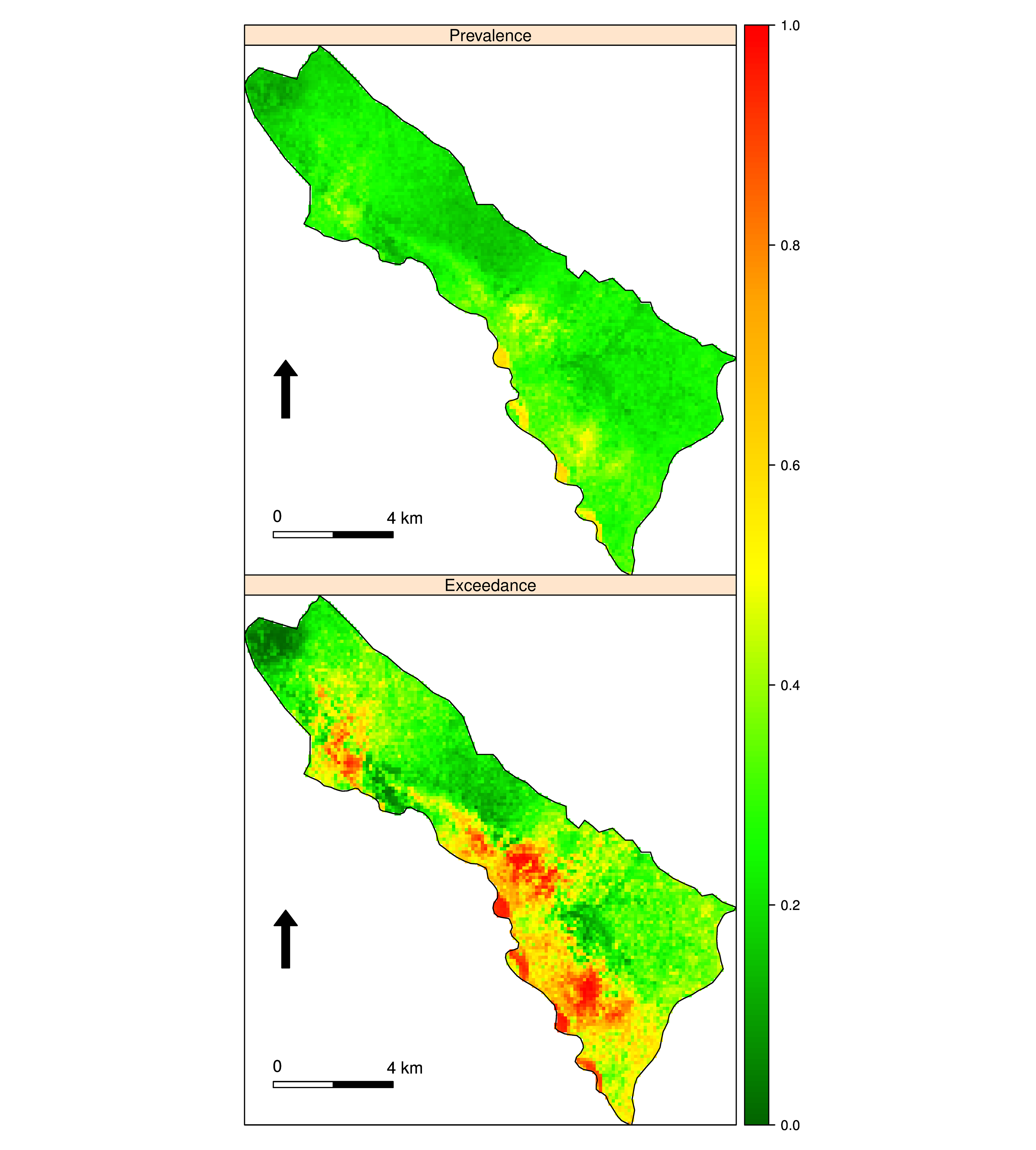

Supplement: S2 Fig — The top panel show malaria prevalence in children 6–59 months in focal area C. The bottom panel shows the map of exceedance probabilities P(x; 0.3) for the Bayesian prediction. (TIF) [file pone.0172266.s002.tif]

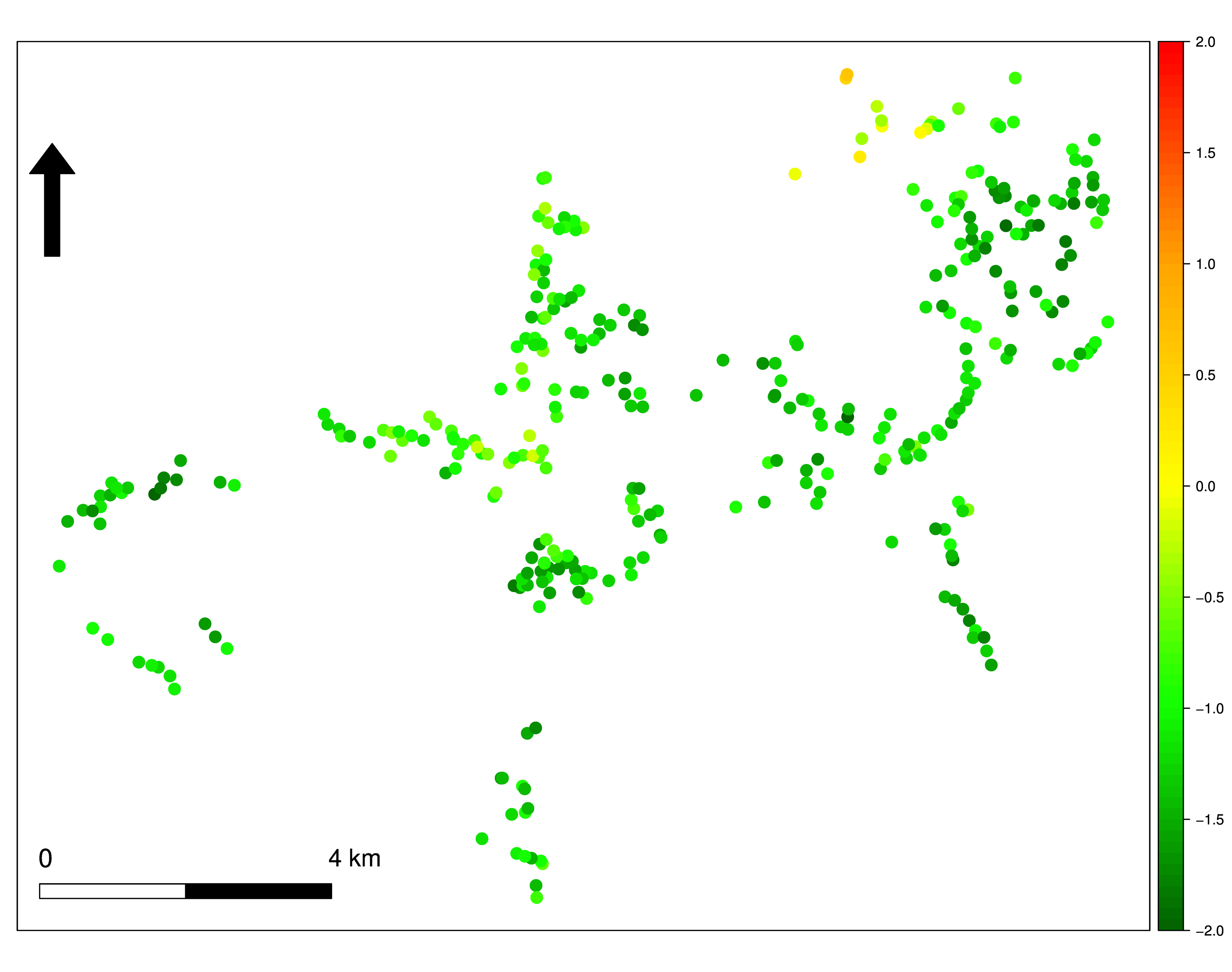

Supplement: S3 Fig — Contributions of the linear regression and of the unexplained spatial variation to the predicted log-odds of malaria prevalence in children 6–59 months at each of the observed locations in focal area A. (TIF) [file pone.0172266.s003.tif]

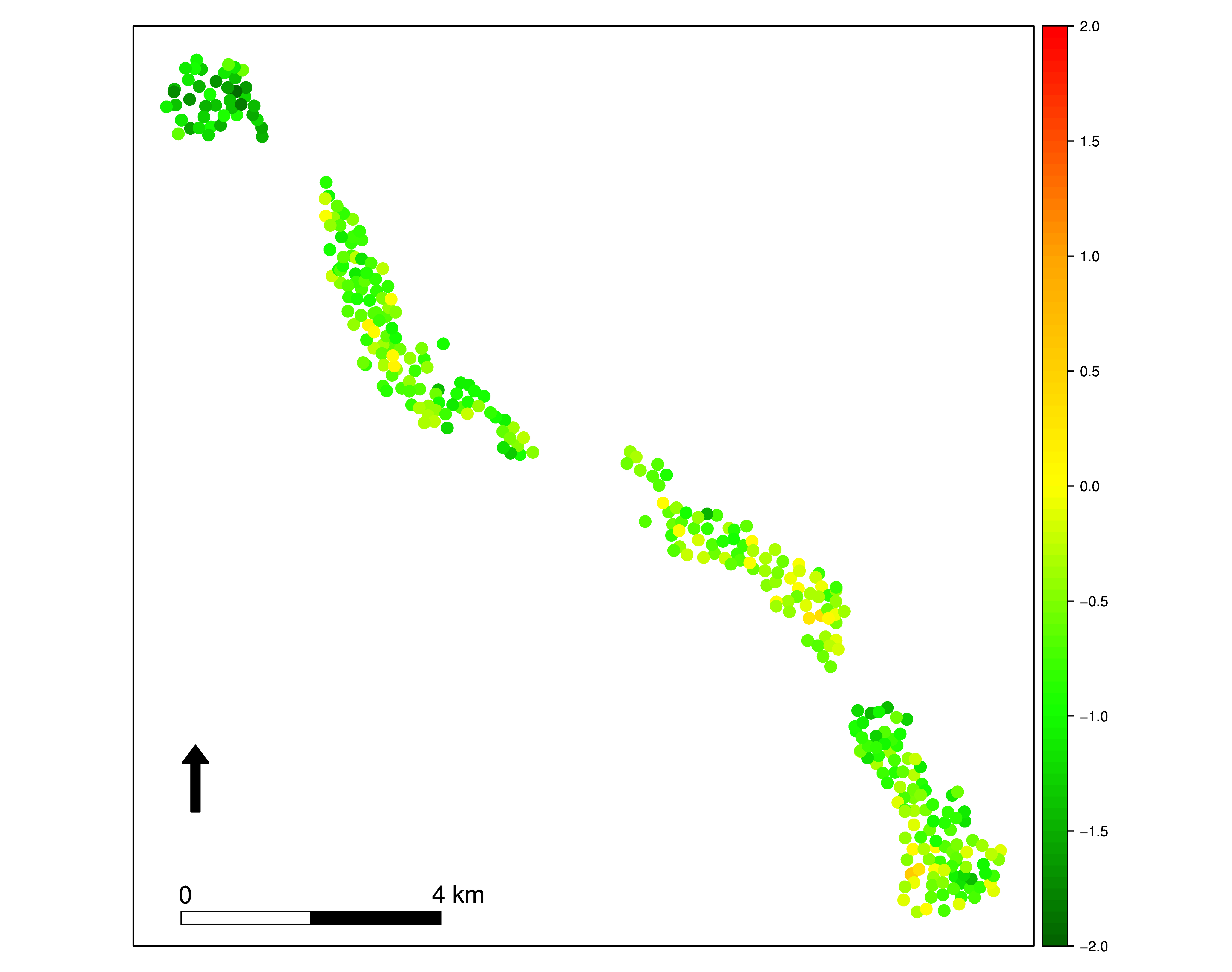

Supplement: S4 Fig — Contributions of the linear regression and of the unexplained spatial variation to the predicted log-odds of malaria prevalence in children 6–59 months at each of the observed locations in focal area A. (TIF) [file pone.0172266.s004.tif]
