# Supplementary material for: Adaptive geostatistical sampling enables efficient identification of malaria hotspots in repeated cross-sectional surveys in rural Malawi
Source: PLoS One. 2017 Feb 14;12(2):e0172266. doi: 10.1371/journal.pone.0172266 (PMC5308819; doi:10.1371/journal.pone.0172266)
Supplement: S1 Table — ITN: insecticide treated bed nets, NDVI: normalized difference vegetation index, SES: socio-economic status. (DOCX) [file pone.0172266.s006.docx]

|  | Estimate | Std. Error | Z value | P value |
| --- | --- | --- | --- | --- |
| (Intercept) | 0.19176 | 0.22873 | 0.84 | 0.40181 |
| SES | -0.09046 | 0.02203 | -4.11 | <0.001 |
| ITN | -0.25797 | 0.06732 | -3.83 | <0.001 |
| Age | -0.45688 | 0.06183 | -7.39 | <0.001 |
| Elevation | -0.00123 | 0.00018 | -6.71 | <0.001 |
| NDVI | 1.58551 | 0.51214 | 3.10 | 0.002 |
